# Intratumoral Microbiota: Insights from Anatomical, Molecular, and Clinical Perspectives

**DOI:** 10.3390/jpm14111083

**Published:** 2024-10-31

**Authors:** Claudia Lombardo, Rosanna Fazio, Marta Sinagra, Giuseppe Gattuso, Federica Longo, Cinzia Lombardo, Mario Salmeri, Guido Nicola Zanghì, Carla Agata Erika Loreto

**Affiliations:** 1 Department of Biomedical and Biotechnological Sciences, University of Catania, 95123 Catania, Italy; claudia.lombardo@unict.it (C.L.); rosannafazio34@gmail.com (R.F.); marta.sinagra@live.it (M.S.); cinzialombardo@hotmail.com (C.L.); msalmeri@unict.it (M.S.); carla.loreto@unict.it (C.A.E.L.); 2 Department of General Surgery and Medical-Surgical Specialties, Policlinico-Vittorio Emanuele Hospital, University of Catania, 95123 Catania, Italy; gzanghi@unict.it

**Keywords:** intratumoral microbiota, microbiota, microbiome, anatomical microbial distribution

## Abstract

The human microbiota represents a heterogeneous microbial community composed of several commensal, symbiotic, and even pathogenic microorganisms colonizing both the external and internal body surfaces. Despite the term “microbiota” being commonly used to identify microorganisms inhabiting the gut, several pieces of evidence suggest the presence of different microbiota physiologically colonizing other organs. In this context, several studies have also confirmed that microbes are integral components of tumor tissue in different types of cancer, constituting the so-called “intratumoral microbiota”. The intratumoral microbiota is closely related to the occurrence and development of cancer as well as to the efficacy of anticancer treatments. Indeed, intratumoral microbiota can contribute to carcinogenesis and metastasis formation as some microbes can directly cause DNA damage, while others can induce the activation of proinflammatory responses or oncogenic pathways and alter the tumor microenvironment (TME). All these characteristics make the intratumoral microbiota an interesting topic to investigate for both diagnostic and prognostic purposes in order to improve the management of cancer patients. This review aims to gather the most recent data on the role of the intratumoral microbiota in cancer development, progression, and response to treatment, as well as its potential diagnostic and prognostic value.

## 1. Introduction

**Figure 1:**
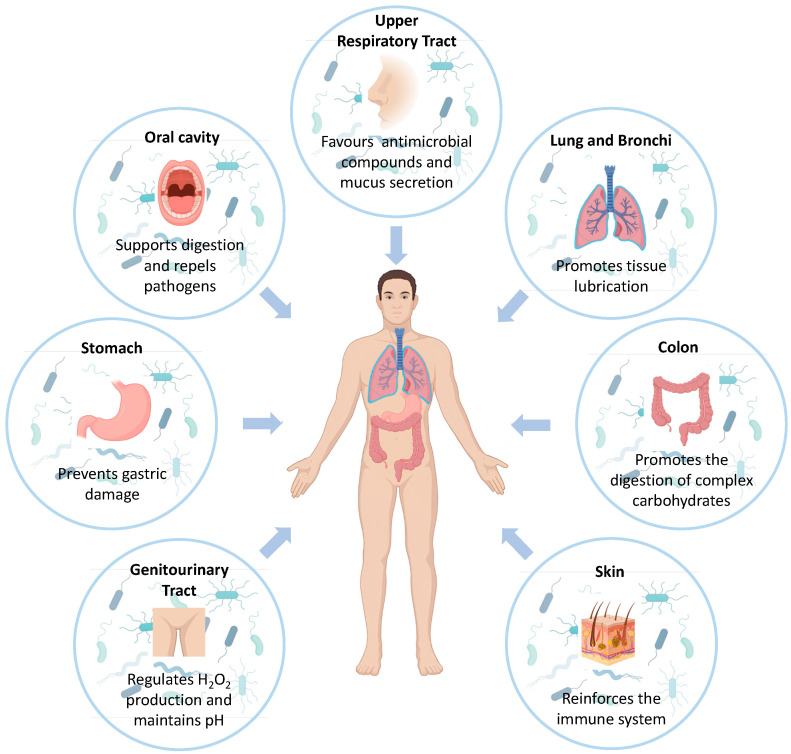
Different anatomical sites of human microbiota and its functions. In the upper respiratory tract, the microbiota contributes to the production of mucus and antimicrobial chemicals. In the lungs and the bronchi, the microbiota promotes tissue lubrication. In the colon, microbiota aids the digestion of complex carbohydrates. The skin microbiota strengthens the immune system. In the genitourinary tract, microbiota maintains pH and regulates H_2_O_2_ production. The gastric microbiota prevents stomach damage. The oral microbiota promotes digestion and limits pathogen ingestion.

The human microbiota consists of a variety of microorganisms that live in the human body. These microorganisms are mostly represented by bacteria but also include viruses, fungi, and protozoa. Microbes colonize different sites of the human body, such as the skin, mucous membranes, and the digestive tract, especially the large intestine. In each of these areas, microbes play important physiological and metabolic functions (Figure 1) [1]. A balanced state of microbiota, defined as eubiosis, consists of a preponderance of potentially beneficial species. In contrast, a state of dysbiosis occurs when the stable equilibrium is lost, and pathogens prevail over beneficial species [2].

The microbial colonies that compose the human microbiota influence important physiological functions such as the metabolism of nutrients, the processing of drugs and xenobiotic substances, and the regulation of the immune system, and can also influence tumorigenesis, tumor progression, and therapeutic response [1,3]. As these microbial communities vary among different anatomical sites, complex host–microorganism interactions were established, making microbiota a potential determinant of disease development, including cancer [1,2,3,4]. Recent epidemiological data have demonstrated that the development of approximately 20% of cancers worldwide is associated with microbial dysbiosis [4]. According to these data, the International Association for Cancer Registries (IACR) has recognized 11 microbial species as human carcinogens [5]. Among these “oncomicrobes”, *Helicobacter pylori*, *Fusobacterium nucleatum*, *Escherichia coli*, *Bacteroides fragilis*, and *Porphyromonas gingivalis* are part of the human microbiota; therefore, the abundance of these harmful bacteria should be carefully assessed to predict the risk of development of cancer [4].

The understanding of the role of human microbiota in the development of diseases, particularly cancer, has expanded significantly. While it was previously thought that cancerous tissues were sterile, recent research has revealed the presence of microbes within the tumor bulk, forming what is known as the intratumoral microbiota [6]. The first findings on tumor microbial infiltration both refer to the role of *F. nucleatum* and date back to 2013 and 2017, respectively. More in detail, in 2013, Kostic AD and colleagues demonstrated that *F. nucleatum* is enriched in adenomas and adenocarcinomas as well as in the tumor microenvironment (TME) of patients with cancer postulating and demonstrating in mice models that this microbe can contribute to colorectal (CRC) tumorigenesis [7]. In 2017, Park HE and collaborators analyzed the presence of the 16S ribosomal RNA gene DNA sequence in 160 surgically resected MSI-H CRC tissues in three different clusters of patients with high, low, or negative abundance of *F. nucleatum* DNA. The authors also demonstrated that the intratumoral infiltration of *F. nucleatum* was associated with the clinical–pathological and molecular features of patients, including the microsatellite instability (MSI) status [8]. These two first studies completely changed the paradigm according to which tumor tissues were sterile, and microbiota was a separate entity, paving the way for several studies aimed at investigating the role of intratumoral microbiota in cancer.

Specifically, a growing body of studies is demonstrating the role of intratumoral microbiota in the modulation of tumor development, TME, and therapeutic response. Indeed, some microbes can directly cause DNA damage; others can induce the activation of proinflammatory responses or oncogenic pathways. Starting from these observations, in the present manuscript, we want to review the latest reports about the association of intratumoral microbiota with different clinical–pathological features of cancer patients, thus arguing its possible role as a prognostic biomarker.

## 2. Site-Specific Human Microbiota

### 2.1. Gut Microbiota

Gut microbiota is the most widely studied microbial ecosystem, consisting of more than 400 different microbial species that live in the gastrointestinal tract. To date, approximately 50 bacterial phyla have been described, of which the predominant ones are the Bacteroidetes and Firmicutes, while other phyla present a smaller abundance, including Actinobacteria, Verrucomicrobia, Proteobacteria, Cyanobacteria, and Fusobacteria. Firmicutes, composed of more than 200 genera, including Lactobacillus, Mycoplasma, Bacillus, and Clostridium, are very common in the intestinal microbiota [9].

Generally, the microorganisms present in the gut are considered “good” as they are involved in the metabolism of nutrients and drugs, preventing the colonization of pathogens and stimulating the immune system that co-evolves together with the healthy microbiota [10].

The composition of human gut microbiota varies depending on many factors, such as genetic background, environmental factors, type of delivery (natural or cesarean) and breastfeeding received, diet, habits, and behavior [11].

As previously mentioned, dysbiosis is characterized by a decrease in the amount and diversity of microorganisms that constitute a healthy intestinal microbiota. In particular, dysbiosis is characterized by the increase in the abundance of bacterial species that have the potential to cause disease (e.g., *Escherichia coli*, Enterobacteriaceae, *Clostridium difficile*, etc.), usually inhibited by the predominance of healthy bacteria (in particular, *Lactobacilli*) and the pH status of the intestinal environment [12].

Recent studies have demonstrated the association existing between several cancers and microbial dysbiosis, highlighting the potential predictive value of the presence of specific intestinal microbes to predict the development of tumors and the prognosis of patients. Furthermore, intestinal microbes have also been associated with other bowel diseases like inflammatory bowel disease (IBD) and irritable bowel syndrome (IBS). Other findings demonstrated the association between gut microbiota and metabolic disorders, allergic disease, and neurodevelopmental disorders [10]. Finally, the microbiota is often related to colorectal cancer (CRC), and the identification of harmful or protective microorganisms and their metabolites in patients is gaining great relevance for the diagnosis and treatment of CRC [13].

### 2.2. Oral Microbiota

The oral cavity is one of the most exposed surfaces of the body, and it is colonized by a diversified microbiota. In this site, the oral microbiota plays an important defensive role and acts toward respiratory tract-associated pathogens associated with oral and respiratory infections. *Streptococcus salivarius* is the most found strain in the oral cavity responsible for the maintenance of oral cavity eubiosis [14]. Besides protective bacteria, *Porphyromonas gingivalis* and *F. nucleatum* are oral taxa that have carcinogenic potential as they can induce several mechanisms such as the loss of apoptosis, the promotion of cell proliferation and invasion, the stimulation of proinflammatory factors and the accumulation of carcinogens responsible for the neoplastic transformation of the normal mucosa [15]. Usually, oral cavity dysbiosis is associated with a predominance of harmful bacteria, including *Moraxella catarrhalis*, *Haemophilus influenzae*, and other *Streptococcus species*, such as *S. pneumoniae* and *S. pyogenes*. Oral cavity dysbiosis is associated with both local inflammatory conditions (dental caries, periodontitis, gingivitis, and abscesses) and numerous systemic diseases [15]. Despite the dense microbial colonization in the oral cavity, acute infections are quite rare due to the balance maintained by the interaction between bacteria and the immune system of the human host [15]. Recently, a close association between the oral microbiota and the development of precancerous and cancerous oral lesions has been found [16,17]. Usually, *Tannerella forsythia*, *P. gingivalis*, and *Treponema denticola* are not found in healthy oral cavities, while *Aggregatibacter actinomycetemcomitans*, *P. gingivalis*, *T. forsythia*, *T. denticola*, *Prevotella intermedia*, *P. nigrescens*, *Parvimonas micra*, *Campylobacter rectus*, and *F. nucleatum* are a group of pathogens defined as periodontal. Periodontitis patients have been shown to have a 2- to 5-fold higher risk of contracting any cancer than healthy controls, particularly oral squamous cell carcinoma (OSCC) [18]. Moreover, the World Health Organization has defined some oral lesions as potentially malignant as associated with the development of OSCC [19]; these include oral lichen planus (OLP), a chronic inflammatory mucosal disease of unknown origin [17]. Patients with OLP often present oral dysbiosis with an abundance of *Porphyromonas*, *Solobacterium*, and *P. melaninogenenica*, and to a lesser extent with the presence of *Haemophilus*, *Corynebacterium*, *Cellulosimicrobium*, and *Campylobacter* [20].

### 2.3. Vaginal Microbiota

The vaginal mucosa is a more complex site for bacteria growth as it is characterized by an acidic environment. Compared to the intestinal or oral microbiota, the vaginal microbiota has a completely different composition in terms of bacterial species. Several species of *Lactobacilli* are predominant in normal vagina, defending this environment by secreting antibacterial substances, including defensins and cytokines. The amount of *Lactobacillus iners* increases in case of dysbiosis [21]. A reduction in these microorganisms may lead to several negative outcomes, such as a higher susceptibility to sexually transmitted infections (STIs), risk of preterm birth, spontaneous miscarriage, and pelvic inflammation disease [22].

An altered vaginal microbiota can be the cause of various diseases, such as bacterial vaginosis (BV) [23] or Aerobic Vaginitis [24], genetic traits, underlying health conditions (such as cardiometabolic, neuroendocrine, and immune-inflammatory disorders), and environmental influences. For instance, cigarette smoking has been closely linked to a higher prevalence of bacterial vaginosis (BV) and disruptions in the vaginal microbiome [25]. Cervical cancer is the most common cancer linked to HPV. Research has shown that a vaginal microbiome not dominated by Lactobacillus species is linked to a greater likelihood of HPV infection and its persistence. Furthermore, in cases of BV, the reduction in *Lactobacillus* spp. and the overgrowth of certain anaerobic bacteria have been connected to an elevated risk of contracting HPV [26]. *Sneathia sanguinegens*, *Anaerococcus tetradius*, and *Peptostreptococcus anaerobius* are enriched in the vaginal microbiota of high-grade dysplasia patients [27]. Another study showed that *Sneathia sanguinegens* and *Fusobacterium* spp. were present only in patients with cervical precancerous lesions or carcinoma and not in healthy patients [28]. In addition to BV-associated bacteria, other bacteria responsible for vaginal dysbiosis, such as *Streptococcus agalactiae* and *Clostridium*, have been detected in HPV-positive women or women with precancerous and cancerous lesions. In addition, the risk factors associated with intestinal and vaginal dysbiosis, i.e., obesity, inflammation, hormonal imbalance, and estrogen therapy after menopause, are the same that lead to a higher risk of endometrial cancer, supporting the notion that microbiota alteration is associated with the etiology of endometrial cancer [25]. Bacteria found in women with gynecological hyperplasia and cancer are *Atopobium*, *Porphyromonas*, *Dialister*, *Peptoniphilus*, *Ruminococcus*, *Anaerotruncus*, *Anaerostipes*, *Treponema*, *Bacteroides*, and *Arthrospira* [29]. Sexually transmitted pathogens causing chronic infections and inflammation have also been linked to the onset of ovarian tumors, including *Brucella*, *Mycoplasma*, and *Chlamydia* spp. [30,31,32].

### 2.4. Respiratory Tract Microbiota

Although previously considered a sterile site, research has revealed that the lungs harbor distinct microbial communities [33]. The lung microbiota is mainly composed of two bacterial phyla, Bacteroidetes and Firmicutes [34,35]. However, *Prevotella* and *Veillonella* are also found in healthy lungs [36]. In the lower respiratory tract, *Megasphaera*, *Streptococcus*, *Pseudomonas*, *Fusobacterium*, and *Sphingomonas* are predominant [34,36]. The pulmonary microbiota may also be associated with tumors and other diseases. Compared to healthy lung tissue, lung tumor is enriched in bacterial families such as *Herbaspirillum* and *Sphingomonadaceae* [37].

Furthermore, the alteration of lung microbiota may lead to a state of chronic inflammation, altering the immune microenvironment and supporting tumor progression. Also, in other lung diseases, such as chronic obstructive pulmonary disease and cystic fibrosis, dysregulation of lung microbiota was found [38]. Loss of bacterial diversity is common in patients presenting respiratory diseases [39]. A prevalence of *Veillonella* and *Rothia* and a concomitant reduction in *Streptococcus* spp has been found in metastatic squamous cell lung carcinoma patients compared to non-metastatic ones [40]. Some authors have also proposed the detection of *Veillonella* and *Megasphaera* in lung samples (e.g., bronchoalveolar lavage samples) as predictive biomarkers for the diagnosis of lung cancer [39].

### 2.5. Skin Microbiota

Another anatomical site with an abundant microbiota is the skin. The main genera of commensal bacteria in normal skin are *Corynebacteria*, *Propionibacteria*, and *Staphylococcus*. However, the composition of the skin microbiota depends on its location [41]. Several studies have shown that skin tumors are often subject to the action of different microorganisms, such as bacteria of the genus *Acinetobacter*, *Actinomyces*, *Corynebacterium*, *Enterobacter*, and *Streptococcus* [42]. The most common taxa in melanoma could include *Paracoccus marcusii* and *Staphylococcus aureus* [6]. The presence of *Staphylococcus aureus* was also found in squamous cell carcinoma, and it is closely associated with carcinogenesis [43].

## 3. The Intratumoral Microbiota

Starting from the well-known notion that microorganisms colonize different anatomical sites, in recent years, researchers have begun to question if bacteria or other microorganisms may infiltrate the tumor tissue, thus influencing tumor initiation and progression as well as cancer immune escape and immune response. Therefore, the concept of intratumoral microbiota has emerged, referring to the microbiota localized within the tumor bulk, and some studies have investigated the presence of specific microbial signatures in different tumors, including colorectal cancer, pancreatic cancer, lung cancer, breast cancer, etc. [44].

The precise characterization of intratumoral microbiota composition is difficult as the microbial abundance in tumors is extremely low, and the detection methods have a low sensitivity rate. However, with the development of next-generation sequencing and highly sensitive technologies, it is now possible to explore the composition of the intratumoral microbiota and unveil its role in tumorigenesis and tumor progression [45]. A recent study aimed at evaluating the composition of intratumoral microbiota in 516 normal samples and 1010 tumor samples obtained from seven types of cancer (breast, lung, ovarian, melanoma, bone, and brain cancer) showed the presence of specific microbial signatures depending on the type of tumor [6].

### 3.1. Mechanisms Leading to the Formation of the Intratumoral Microbiota

Before describing the role of intratumoral microbiota in tumor initiation and progression, it is important to understand the mechanisms underlying the formation of intratumoral microbiota. The precise mechanisms responsible for the migration of microorganisms within tumor mass are still not fully understood; however, some mechanisms have been postulated and partially confirmed, including the disruption of mucosal barriers, the migration from normal adjacent tissue (NAT), and migration from blood or lymphatic vessels [46]. The understanding of these mechanisms will be helpful to shed light on the complex relationships between host cells and microbial inhabitants within the tumor milieu, offering insights into novel avenues for cancer diagnosis, prognosis, and treatment strategies.

#### 3.1.1. Intratumoral Microorganisms Migrating from Disrupted Mucosal Barriers

Intratumoral microorganisms are most likely to be found in tumors arising from mucosal sites [47]. Usually, the inner organs and tissues of the host are separated from the microbiota by an intact mucosal barrier, which prevents microorganisms from penetrating the tissue and causing disease. However, tumor cells and cells of the TME may alter the functionality of the mucosal barrier, disrupting its integrity and determining localized inflammation and immune imbalance that may favor microbial infiltration. It was demonstrated that gut microbiota can infiltrate colorectal cancer lesions and act as a complex and independent intratumoral microbiota [48]. Due to the proximity to the intestinal tract, also pancreatic cancer may interact with the gut microbiota, contributing to the development of an independent pancreatic intratumoral microbiota resulting from the disruption of the mucosal barrier and the consequent migration of bacteria within the tumor bulk [49]. The mechanism by which microbiota enters from distant sites to tumor bulk has yet to be elucidated [47,50]. Some factors involved in the migration of bacteria via disrupted mucosal barriers, like the alteration of zonulin expression, have been postulated and partially proved [51,52].

#### 3.1.2. Intratumoral Microbes from Normal Adjacent Tissue (NAT)

The presence of intratumoral microbiota was also observed in tumors arising in distant organs compared to the normal localization of microbiota (i.e., oral cavity, large intestine, and vagina). For instance, intratumoral microorganisms were found in breast cancer, a tumor that does not originate from mucosal sites or takes origin from the circulatory system. This suggests that the migration of microorganisms is not only mediated by altered mucosal barriers or microorganisms present in the bloodstream [47].

In their study, Nejman D et al. showed how NATs could be potential sources of tumor microbiota. The authors suggested that intratumoral microbiota could originate from NATs due to the fact that the same species are detected within tumor tissues and NATs; however, no clear demonstrations of the same origin have been obtained yet, highlighting the need for further investigations on this topic [6]. In this context, several factors associated with the formation of intratumoral microbiota from NATs have been proposed, including hypoxia, inflammation, and altered immune responses. This altered environment, in association with tumor-associated molecular changes, facilitates the recruitment and proliferation of microbial populations from neighboring normal tissues [47]. Furthermore, hypoxia tumor-derived factors, including metabolic byproducts and secreted molecules, may create a selective advantage for certain microbial species, further shaping the composition of the intratumoral microbiota with slight differences compared to the NATs microbiota [53].

#### 3.1.3. Intratumoral Microbes from the Circulatory System

Microbiota can colonize both the tumor tissue and the TME by hematogenous dissemination infiltrating the tumor mass from damaged vessels. Microbiota transfer is facilitated by blood and lymphatic flows and by the internal canals in the gut [54]. The main sources of intratumoral microbiota are the oral cavity, the respiratory tract, and the digestive system; therefore, microorganisms localized in these districts can be easily disseminated in other parts of the body through the lymphatic or hematogenous circulation [55].

### 3.2. Intratumoral Microbiota in Different Tumors

To date, the presence of bacteria in tumors is firmly established in different tumors (Table 1); nevertheless, it remains unclear whether their presence favors tumor progression, influences the immune response, or only facilitates the survival of bacteria in other sites. It was already demonstrated that 16S rRNA and bacterial lipopolysaccharide (LPS) can be detected in all tumors [6]. In particular, breast cancer appears to be the most microbial-enriched tumor, with about 16 different bacterial strains, while less than nine bacterial strains were detected in other types of tumors. The most prevalent species detected among the different types of tumors belonged to Proteobacteria and Firmicutes. The phylum Actinobacteria, including the families Corynebacteriaceae and Micrococcaceae, is the most prevalent in tumors not affecting the gastrointestinal tracts [6].

#### 3.2.1. Breast Cancer Intratumoral Microbiota

Breast microbiota is fundamental in regulating women’s health and disease. Numerous studies have characterized and compared the composition of breast microbiota observed in tumor and healthy tissues. In the Canadian population, *Bacillus*, *Acinetobacter*, *Enterobacteriaceae*, *Pseudomonas*, *Staphylococcus*, *Propionibacterium*, *Comamonadaceae*, *Gammaproteobacteria*, and *Prevotella* were predominant in breast cancer samples than in normal adjacent tissue, while *Enterobacteriaceae*, *Staphylococcus*, *Listeria welshimeri*, *Propionibacterium*, and *Pseudomonas* were the most abundant in the tumor samples obtained from an Irish cohort. The prevalence of *Proteobacteria* and *Firmicutes* (particularly the class Bacilli) over other taxa could be a consequence of the adaptation of the host microbiota to the fatty acid environment typical of breast tissue [56]. According to a study by Xuan et al., *Methylobacterium radiotolerans* is relatively enriched in tumor tissue, whereas *Sphingomonas yanoikuyae* is relatively enriched in paired normal tissue. These bacteria can contribute to the regulation of the breast microenvironment by promoting or inhibiting the local immune system. In the case of dysbiosis, a significant reduction in microbial abundance may lead to a decrease in immune cell activity. In particular, the reduction in *S. yanoikuyae* was associated with the loss of function of immune cells, creating an environment predisposing to breast tumorigenesis [57]. Some of the relatively more abundant bacteria in breast cancer patients (*E. coli*, *S. epidermidis*, *B. cereus*) can induce DNA double-strand breaks or have other carcinogenic effects [58]. It was also demonstrated that the evaluation of the bacterial load can be used as a potential marker to evaluate disease progression and tumor staging since different concentrations of bacteria are detected at different tumor stages. Furthermore, a reduction in the bacterial abundance observed in healthy individuals could be related to a reduced risk of breast cancer [57]. Chemotherapy alters the breast cancer microbiome. For instance, neoadjuvant chemotherapy has been seen to lead to elevated levels of *Pseudomonas* and decreased levels of *Prevotella* in breast cancer. Moreover, the prevalence of *Brevundimonas* and *Staphylococcus* observed in cancer patients was also associated with the development of metastatic disease [59].

A recent study by Laborda-Illanes and colleagues (2024) also showed microbial diversity in the intratumoral microbiota of metastatic and non-metastatic patients, with the latter showing the predominancy of bacteria with anti-inflammatories, including *Bifidobacterium adolescentis*, *Bifidobacterium longum*, *Lactobacillus iners*, and *Faecalibacterium prausnitzii* [60].

These data suggest that intracellular microbes are fundamental for the regulation of metastasis formation by influencing the cell cytoskeleton and cell viability in case of oxidative and mechanical stress. Additionally, as supported by the theory of the origin of the intratumoral microbiota from the circulatory system, it can be hypothesized that during the spreading of tumor cells in distant anatomical sites, intracellular microorganisms can also be disseminated through the circulatory system, thus migrating in distal organ [61].

#### 3.2.2. Lung Cancer Intratumoral Microbiota

A predominance of *Actinobacteria*, *Proteobacteria*, *Firmicutes*, and *Bacteroidetes* was observed in tumor tissue and adjacent normal mucosa obtained from non-small cell lung carcinoma (NSCLC) patients. In advanced tumors, the presence of *Pseudomonas*, *Burkholderia*, and *Aquabacterium* was lower, while *Corynebacterium*, *Sphingomonas*, *Streptococcus*, *Neisseria*, *Halomonas*, *Kocuria*, *Parvimonas*, and *Rothia* were more present than in Stage I-II tumors [62]. In squamous cell carcinoma, *Acidovorax* was found to be enriched in smokers. This finding suggests that smoking, besides favoring tumorigenesis, may provide a favorable environment for the growth of *Acidovorax* spp. and similar strains, which can thrive in starving environments such as the lungs. Consequently, it is possible to speculate that lung epithelial cells exposed to tobacco smoke and/or TP53 mutations are infiltrated by microorganisms that could act as promoters of lung tumorigenesis [63].

A recent study performed on early-stage non-smoker lung cancer patients showed that intratumoral microbiota is similar to that of NAT but differs from the composition observed in lung samples obtained from healthy donors. Early-stage lung cancer patients presented a lower abundance of Proteobacteria and an increased presence of Actinobacteria compared to healthy donors. In particular, *Bacillus thuringiensis*, *Acinetobacter baumannii*, *Mycobacterium tuberculosis*, and *E. coli* were the most abundant taxa in patients with early-stage tumors [64].

It has been validated that microbiota composition has an important role in lung cancer development and therapeutic response, particularly in patients treated with immunotherapy. Some research demonstrated that antimicrobial agents and proton pump inhibitors (PPIs) may induce changes in the relative abundance of microbial genera and taxa. The modulation of both microbiota composition and immune components suggests that the alteration of the host microbiome could affect the efficacy of ICIs in NSCLC patients. These findings were also confirmed by preliminary observations on the use of probiotics in these patients that ameliorate some of the side effects of patients’ treatment [65]. Lastly, lung microbiota also influences the efficacy of chemotherapy, targeted therapies, and immunotherapy efficacy [66].

#### 3.2.3. Cutaneous Melanoma Intratumoral Microbiota

Human cancers, including melanoma, can be colonized by several bacteria. A study conducted in melanoma patients found the presence of *Propionibacterium*, *Staphylococcus*, and *Corynebacterium* in the cancer samples obtained during surgery [67]. The presence of bacterial-derived HLA peptidomic signatures has been observed in the tumors of melanoma patients. The bacterial HLA-I and HLA-II peptides can be internalized by both antigen-presenting cells and melanoma cells. Since bacterial antigens are not recognized as self, these can be targeted with immunotherapy [68,69]. However, the selection of bacterial antigens for immunotherapy should be highly accurate by favoring bacterial species known to have positive effects on the immune system [42,70]. In a research performed by Mizuhashi et al. using culture methods and not the molecular sequencing of 16S rRNA, the authors observed a clinically significant association between *Corynebacterium* and the progression of acral melanoma [71]. By analyzing the intratumoral microbiota of melanoma and patients’ response who responded to ICIs, an enrichment of *Clostridium* was also observed [6]. Zhu et al. observed an association between intratumor bacteria and infiltrated CD8+ T cells. Specifically, *Algibacter* and *Epilithonimonas* showed a negative correlation with CD8+ T cells, while *Lachnoclostridium*, *Gelidibacter*, *Flammeovirga*, *Acinetobacter*, and *Tropicibacter* had a positive correlation with infiltrated CD8+ cells. Moreover, a correlation between chemokines for CD8+ T and intratumoral bacteria was also found. Surprisingly, most genera that were positively correlated with tumor-infiltrating CD8+ T cells showed a positive association with the expression of chemokines like CXCL9, CXCL10, and CCL5, whereas the two genera negatively associated with intratumoral CD8+ T cells also showed a negative association with the same factors. Finally, the presence of intratumoral *Lachnoclostridium* was associated with better overall survival of melanoma patients [72].

#### 3.2.4. Pancreatic Cancer Intratumoral Microbiota

Several studies demonstrated the pathogenetic role of microorganisms and their products in the development and progression of pancreatic cancer. In both tumor samples and adjacent tissue, *Helicobacter* DNA was detected, supporting the involvement of *Helicobacter* spp. in the induction of pancreatic inflammation and, consequently, cancer [73]. *E. coli* and *B. fragilis* can induce pancreatic cancer development by suppressing the immune response [74]. *F. nucleatum*, often observed in colorectal cancer, has also been found in pancreatic tumors [75]. In a study by Mitsushashi et al., *Fusobacterium* was detected in less than 10% of pancreatic ductal adenocarcinoma (PDAC) tissues, and the presence of this microorganism was correlated with lower survival rates in cancer patients [76]. The analysis of 113 human PDAC samples showed that 76% of tumor samples had bacterial DNA. Most of the bacteria detected belonged to Gammaproteobacteria, which are responsible for gemcitabine resistance [77].

The evaluation of the predominance of the three taxa Saccharopolyspora, Pseudoxanthomonas, and Streptomyces and the abundance of *Bacillus clausii* could predict long-term survival in PDAC patients. The composition of the PDAC microbiota was also associated with the host immune response [78]. The presence of infiltrating microorganisms can increase pancreatic cancer response to the immune system with good antitumor effects. In addition, intratumoral microbiota can modulate pancreatic cancer TME by promoting M1 macrophage differentiation and reducing MDSCs. This leads to the activation of CD8+ T cells and the differentiation of CD4+ T cells in TH1 lymphocytes, thereby increasing the expression of Programmed Cell Death Protein 1 (PD-1) and responsiveness to immunotherapy [55]. A study found an association between the incidence of pancreatic cancer and the composition of the salivary microbiota. In particular, it was found that *Aggregatibacter actinomycetemcomitans*, the oral *P. gingivalis*, and circulating antibodies against *P. gingivalis* are strongly associated with a high incidence of pancreatic cancer [79]. In addition, oral microbiota carrying peptidyl arginaminase (PAD), including *Treponema denticola*, *Tannerella forsythia*, and *Prevotella intermedia*, were observed in p53-mutated tumor tissue. Therefore, oral dysbiosis can influence the pancreatic cancer-specific intratumoral microbiota [55]. In addition, tumor-infiltrating microorganisms observed in pancreatic ductal adenocarcinoma (PDAC) often have an intestinal tract origin, thus supporting the theory of intratumoral microbiota formation from disrupted mucosal barriers. These bacteria reach the pancreas through the pancreatic duct, remodeling the TME and inducing innate and adaptive immunosuppression [80]. Furthermore, in pancreatic cancer animal models, the gut microbiota consisting of *Proteobacteria*, *Bacteroidetes*, and *Firmicutes* produced an immunosuppressive environment by altering the composition of cytokines and activating T cells in the TME, thereby promoting tumor progression [81]. More recently, it was proposed to concomitantly analyze the fecal and intratumor microbiota of pancreatic cancer patients to better profile their prognosis; however, these preliminary findings need confirmation in appropriate clinical settings [82].

#### 3.2.5. Ovarian Cancer Intratumoral Microbiota

Recent studies have demonstrated the presence of intratumoral bacteria colonizing ovarian cancer (OC). Huang and colleagues analyzed similarities and differences in the intratumoral microbiota observed in patients with benign endometrioid borderline ovarian tumors (EBOT) compared to patients with epithelial OC by using high-throughput sequencing methods. The analysis showed that there was significant diversity in the composition of the intratumoral microbiota between EBOT and EOC tissues. In addition, in the cancer group, the presence of *Propionibacterium acnes* was associated with increased OC progression [83]. In another study, researchers utilized advanced 16S rRNA sequencing to investigate the variability of microbiota composition in both ovarian cancer tissues and normal fallopian tubes, revealing a significant reduction in diversity and richness in tumor samples. Specifically, OC intratumoral microbiota showed an increase in the *Proteobacteria/Firmicutes* ratio, which could be associated with OC development [84]. Wang et al. found profound discrepancies between the bacterial compositions observed in cancer patients compared to the control groups. Low concentrations of *Crenarchaeota* and high levels of *Aquificae* and *Planctomycetes* were found in cancer samples, while *Halobacteroides halobius* (14%), *Gemmata obscuriglobus* (11%), and *Methyloprofundus sediments* (10%) predominated in the healthy tissues although the same strains were also observed in cancer tissues in different concentrations (11%, 13%, and 11% respectively) [85]. In a study of 99 OC samples, including 20 matched control samples from adjacent noncancerous tissue and 20 unmatched controls, *Proteobacteria* and *Firmicutes* were found to be significantly increased in OC. *Bacteroidetes*, *Actinobacteria*, *Chlamydiae*, *Fusobacteria*, and *Tenericutes* were found in low concentrations in the cancer samples [32]. Sheng et al. conducted a study in which OC samples were clustered into two subgroups, i.e., immune-enriched and immune-deficient tumors. The infiltration of cytotoxic CD8^+^ T-lymphocytes in OC was significantly associated with a better prognosis. *Acinetobacter baumannii* was significantly enriched in the group of immune-enriched subtypes, contributing to the generation of a proinflammatory microenvironment. *F. nucleatum* was significantly enriched in the immunodeficient phenotype group of patients, and its presence was associated with shorter DFI and PFS [86].

A recent study on 453 OC patients and 68 controls allowed for the identification of a microbial signature predictive of the presence of OC. In particular, Acinetobacteria seems to play a pivotal role as a risk factor in OC development; on the contrary, Proteobacteria were more abundant in the control group. Overall, a total of 197 different species showed a differential abundance in OC compared to normal samples, thus serving as a potential microbial signature for this tumor [87].

#### 3.2.6. Colorectal Cancer Intratumoral Microbiota

Gut microbiota plays an important role in intestinal homeostasis; on the contrary, gut dysbiosis represents a trigger for inflammatory diseases and CRC. A recent study by Cao Y and colleagues identified 18 commensal strains in patients with inflammatory bowel disease that produce small molecules inducing DNA damage. Specifically, in a chemically induced model of colitis-associated cancer, the *Morganella morganii* strain produces a genotoxic indoleamine through a biosynthetic pathway that promotes tumorigenesis [88]. Colibactin produced by Escherichia coli pks+ induces a defined mutational signature detected in CRC. DNA damage is also caused by other bacterial toxins, such as the cytolethal distensible toxin (CDT) present in the human enteric pathogen *Campylobacter jejuni* [48].

Enterotoxigenic *Bacteroides fragilis* (ETBF) strain produces a toxic zinc metalloprotease named BFT, which is responsible for intestinal inflammation and persistent colitis. Furthermore, several epidemiological studies have demonstrated an increased incidence of ETBF infection in CRC patients, suggesting that BFT may play a cause-initiating role in the development of CRC [88]. Another study demonstrated that the oral bacterium *F. nucleatum* could colonize the tumor through hematogenous spread using Fap2 lectin that binds Gal-GalNAc expressed in CRC [89]. Additionally, *F. nucleatum* can also colonize breast cancer with the help of Fap2 lectin. Specifically, bacterial dissemination through the bloodstream can induce the creation of a “premetastatic niche” [90]. In a research conducted on CRC patients, the authors observed that intratumoral *E. coli* can disrupt the intestinal vascular barrier, thus infiltrating the liver and enhancing the formation of a premetastatic niche [91]. *Enterococcus faecalis* is one the most abundant commensal enterococci in human feces, and it was found to be enriched in CRC. *Enterococcus faecalis* is potentially carcinogenic as it can directly damage host DNA through the production of superoxide or a macrophage-induced bystander effect [92]. *Parvimonas micra* and *Peptostreptococcus* spp. are anaerobic Gram-positive commensal cocci that are part of the intestinal or oral microbiota. These genera are enriched in CRC patients, particularly in advanced tumors. *Salmonella* spp., *Clostridium difficile*, *Klebsiella pneumoniae*, *Citrobacter rodentium*, and *Campylobacter jejuni* are other enteric pathogens that may play a role in the onset of CRC colonizing the tumor itself [48].

## 4. Intratumoral Microbiota in Cancer: Tumor Promotion, Tumor Inhibition, and Therapeutic Implications

### 4.1. Role of Intratumoral Microbiota in Cancer Pathogenesis

The formation of distant metastases is recognized as the main cause of mortality in cancer patients. Several processes are necessary for the metastatization of tumors, including the accumulation of mutations, the degradation of the extracellular matrix, and the alteration of the TME [93]. As regards the TME, the microbiota, and the intratumoral microbiota have been recently recognized as pivotal components of the tumor and a possible regulator of metastasis development [94,95].

**Figure 2:**
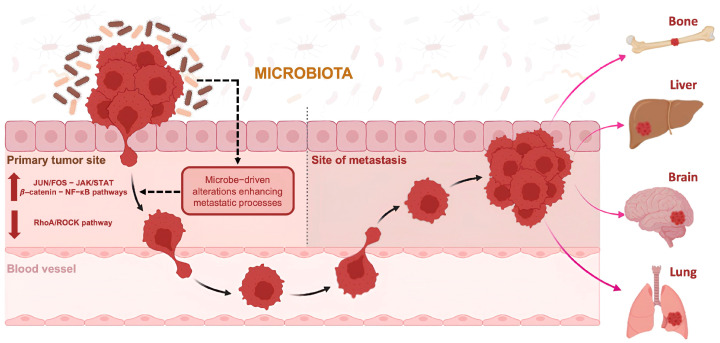
Intratumoral microbiota may facilitate metastasis formation by impairing the mucosal barrier and prompting EMT modifications in cancer cells. The metastatic tumor cells thus invade adjacent tissue as single cells or as clusters of cells and then reach and penetrate blood vessels during intravasation. Finally, tumor cells colonize distal organs, forming metastases in tissues such as the lungs, liver, brain, and bones.

Cancer cell metastasis is largely influenced by cellular traits like EMT status, stem cell flexibility, genetic and epigenetic changes, chromosomal instability, and metabolic adjustments. Additionally, external factors play a key role, such as mechanical forces, immune system responses, ECM degradation, PMNs, and microbiome-induced modifications. In particular, gut microbiota might play a role in transmitting pathogens that confer metastatic properties to tumor cells, inducing molecular changes [96] (Figure 2).

Intracellular bacteria can trigger JUN and FOS family transcription factors, thereby enhancing the expression of genes associated with cancer cell invasion, metastasis, DNA repair, and cellular dormancy. Likewise, invasive bacteria contribute to inflammation by attracting myeloid cells via JAK-STAT signaling and facilitating T-cell exclusion and tumor growth through the release of targeted interleukins and chemokines into the tumor microenvironment [97].

Other studies suggested that intratumoral microorganisms can initiate cancer metastasis. For instance, *Staphylococcus*, *Lactobacillus*, and *Streptococcus* are abundant in the intratumoral microbiota of breast cancer, playing inhibitory effects on the RhoA-ROCK signaling pathway involved in cytoskeleton remodeling. In this way, tumor cells can resist mechanical stress in the bloodstream. In addition, intracellular tumor bacteria may promote tumor metastasis by regulating the activity of the immune cells, as demonstrated in germ-free mice and immunodeficient mice [95].

It was also demonstrated that bacteria can also favor tumor progression by activating tumor-promoting pathways. The activation of the β-catenin signaling pathway can drive malignant cell transformation, given its activity in both host and tumor cells. Additionally, other tumor-intrinsic signaling pathways, such as MAPKs, may be triggered by intratumoral microorganisms through various mechanisms—either by directly stimulating upstream signals or by engaging downstream pathway elements. For example, NF-κB activation leads to the release of cytokines that create a positive feedback loop, fostering chronic inflammation that supports tumor growth [46]. Complement activation contributes to tumor progression by stimulating receptors on the surface of tumor cells. Intratumoral microbiota-induced alterations in signaling pathways can also impact tumor cell metabolism, driving EMT and cell migration. It remains unclear if specific microorganisms target distinct regulatory pathways or if these interactions vary by tumor type. Additionally, it is hypothesized that different microorganisms may act sequentially, affecting signaling at various stages of tumor development and collectively advancing tumor progression [46].

### 4.2. Crosstalk Between Intratumoral Microbiota and TME

**Figure 3:**
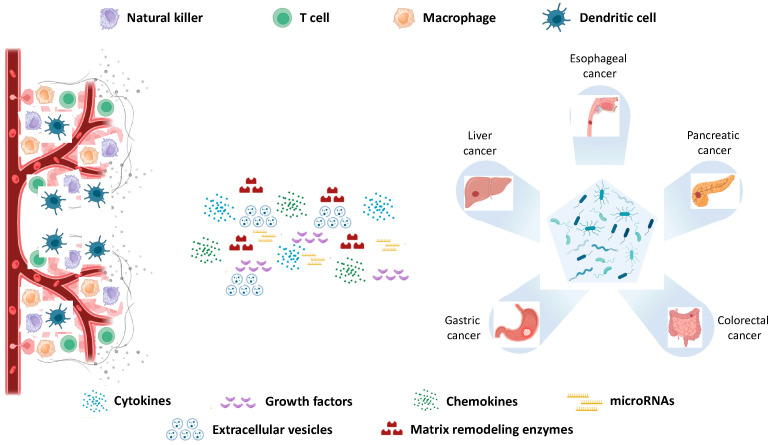
Crosstalk between digestive tumor-associated microbiota and tumor microenvironment (TME). The tumor-infiltrating bacteria can promote immunosuppression or immune reactivation by acting on metabolism and can create a heterogeneous tumor microenvironment to cause cancer initiation and development.

The intratumoral microbiota is heterogeneously distributed among cancer types, representing an important determinant of the TME. Here, intratumoral microbiota can influence the behavior of both normal and tumor cells, modulating the efficacy of antitumor immunity and the migration of tumor epithelial cells. Several studies have revealed that the intratumoral microbiota plays an important role in the modulation of the TME in many types of cancer, especially in epithelial cancers, including lung, skin, cervical, and gastrointestinal tract cancers. Indeed, intratumoral microbiota plays a role in tumor progression and modulates responses to chemotherapy and immunotherapy by influencing inflammation and immune regulation, participating in metabolic processes, and impacting DNA stability [98]. The tumor microenvironment (TME) comprises both proliferating cancer cells and non-malignant components such as immune cells (e.g., microglia, macrophages, lymphocytes), extracellular matrix (ECM) structures, stromal cells, endothelial cells, and bacteria [99]. Within the TME, immune cells are categorized into innate (dendritic cells, innate lymphoid cells, macrophages, myeloid-derived suppressor cells, natural killer cells, and neutrophils) and adaptive (B and T cells) immune cells, with both types capable of promoting either antitumor or pro-tumor effects [100]. The TME and intratumoral microbiota engage in dynamic, reciprocal communication involving cytokines, chemokines, growth factors, matrix-remodeling enzymes, and extracellular vesicles (EVs)—including exosomes, apoptotic bodies, and exosome-derived miRNAs (Figure 3) [100].

Several studies have demonstrated the presence of tumor-infiltrating bacteria also in the TME. These findings support the notion that cancer-associated bacteria may play a role in the modulation of several molecular pathways related to tumor development and progression.

When activated, intratumoral or tumor-associated monocytes regulate macrophage differentiation and polarization by producing interferon-I. These processes also promote cell–cell interaction between natural killer and dendritic cells, creating new balances within the TME [101]. Human bacteria can influence immune cell infiltration in different ways depending on the type of tumor. For instance, in CRC, *Proteus* bacteria (mainly *E. coli*) may infiltrate the liver by crossing the damaged intestinal barrier, promoting the recruitment of immune cells in the liver, thus inducing liver metastasis [45]. In pancreatic cancer, intratumoral bacteria could promote oncogenesis through innate and adaptive immune suppression [81]. Specifically by reducing myeloid-derived suppressor cells, increasing M1 macrophage differentiation, promoting Th1 differentiation of CD4+ T cells, and activating CD8+ T cells [45].

### 4.3. Intratumor-Mediated Tumorigenesis

**Figure 4:**
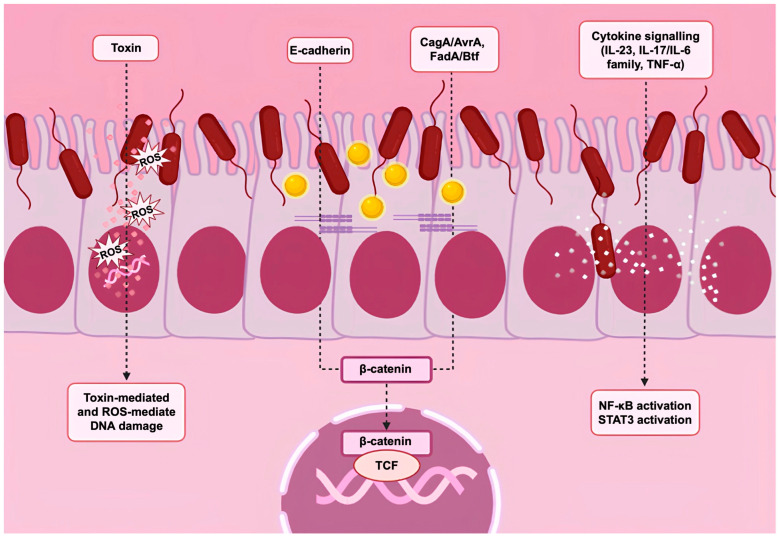
The intratumoral microbiota promotes tumorigenesis and tumor progression through several mechanisms. Many microbes produce compounds like toxins and ROS that can directly cause DNA damage and thus increase mutagenesis. Other microbes promote the activation of oncogenic pathways, such as the β-catenin signaling pathway. For instance, cytotoxin-associated antigen A (CagA) produced by *H. pylori*, *FadA* produced by *F. nucleatum*, Btf produced by *B. fragilis*, and AvrA produced by *S. typhi* interact with E-cadherin or β-catenin enhancing the abnormal proliferation of cancer cells. Intratumoral microorganisms also influence cytokine production, inducing a proinflammatory response and inhibiting the immune response. Finally, the activation of β-catenin induces the nuclear translocation of the β-catenin/T-cell factor/lymphoid enhancer factor family (TCF/LEF) complex, which promotes the transcription of different tumorigenic genes.

Intratumoral microbes play an important role in tumorigenesis and tumor response to treatment through multiple mechanisms (Figure 4). Some microbes can directly cause DNA damage, leading to increased mutations, which in turn can promote tumorigenesis and tumor progression. Particularly in gastrointestinal cancers, the microbiota can cause DNA damage through the production of genotoxic metabolites [102,103]. For example, some members of the Enterobacteriaceae produce colibactin, which causes DNA damage [45,104,105]. The enterotoxigenic strain, *B. fragilis*, can produce toxins that induce DNA damage. In addition, microbes can also cause oxidative/nitrosive DNA damage that leads to tumor development [106].

Many types of microbes have also been shown to promote inflammatory responses and protumoral pathways. Some intratumoral bacteria are able to modulate the release of cytokines, such as IL-6 and TNF-α. The production of IL-17 induced by some intratumoral microbes can cause B-cell infiltration and tumor development [107]. Wnt/β-catenin signaling is another important oncogenic pathway leading to the activation of several oncogenes. Several microbes influence this pathway by directly activating β-catenin or E-cadherin and thus inducing tumorigenesis [108].

The mechanisms by which the intratumoral microbiota promotes tumorigenesis have gained significant attention. Three primary mechanisms are currently recognized: (i) DNA damage induced by secreted metabolites; (ii) activation of oncogenic pathways; and (iii) alteration of the tumor immune microenvironment (Figure 4) [109].

Specifically, several bacteria are able to produce toxic molecules that can induce DNA damage, cell cycle arrest, and genetic instability. The presence of bacteria that produce such compounds within the TME could directly increase mutagenesis in the occupied tissue. Colibactin is a metabolite secreted primarily by B2 strains of the *E. coli* group that can induce genomic instability and double-stranded DNA breaks, thus causing tumor-promoting events [110,111]. A 2020 study further demonstrated the mutagenicity of colibactin, which is closely related to the pathogenicity island pks [105]. Another example is the Bacteroides fragilis toxin (BFT) secreted by *B. fragilis*. BFT causes direct damage to DNA and increases the levels of reactive oxygen species (ROS), further promoting tumorigenesis. These two toxins are both derived from bacteria inhabiting the gastrointestinal tract, favoring the formation of tumors in this site [111].

Microbes also have a tumor-promoting role through the activation of oncogenic pathways. In particular, one of the most influenced pathways is the Wnt/β-catenin signaling pathway, promoting the overexpression of oncogenes, including c-Myc and CyclinD-1. A study performed on gastric and colorectal cancer patients has demonstrated the role of local commensal microorganisms in the activation or modulation of β-catenin [98]. CagA, a protein produced by oncogenic type 1 strains of *H. pylori*, is directly injected into the cytoplasm of host cells, where it induces the aberrant modulation of the β-catenin pathway. CagA-mediated activation of β-catenin results in the upregulation of genes linked to cell proliferation, survival, migration, and angiogenesis, which together drive the development and progression of gastric cancer [108]. *F. nucleatum* produces FadA, a bacterial adhesion protein that binds to host E-cadherin, leading to β-catenin activation. In some human colorectal cancers, enterotoxigenic *B. fragilis* can stimulate E-cadherin cleavage via BTF, also resulting in β-catenin activation. Chronic infections with *Salmonella typhi* strains secrete AvrA, which activates epithelial β-catenin signaling and is associated with hepatobiliary tumors [108].

Finally, tumorigenesis may be promoted by the alteration of the immune microenvironment of tumors promoted by different bacteria. In this context, metabolites produced by microorganisms within the tumor or TME can initiate inflammatory and immunosuppressive responses that foster tumorigenesis and contribute to an immunosuppressive microenvironment that supports tumor progression. In pancreatic cancer, studies have shown that the intratumoral microbiota can inhibit antitumor immunity by altering myeloid-derived suppressor cells (MDSCs), regulatory T cells (Tregs), and the process of antigen presentation. This suppression of immune activity promotes inflammation and generates an immunosuppressive microenvironment that ultimately facilitates pancreatic tumorigenesis [112].

*F. nucleatum* has been shown to modify the tumor immune environment by recruiting tumor-infiltrating myeloid cells, such as dendritic cells (DCs), tumor-associated macrophages (TAMs), MDSCs, and CD11b myeloid cells, thereby enhancing tumor growth. The bacterium’s Fap2 surface protein inhibits natural killer (NK) cells’ ability to destroy tumor cells by blocking the T-cell immunoglobulin and ITIM domain (TIGIT) receptor. Additionally, FapA suppresses T-cell activation by interacting with the same TIGIT receptor, weakening the antitumor response and fostering an immunosuppressive environment [113]. While these effects of *F. nucleatum* have been widely studied in intestinal cancers, further research is needed to clarify the role of intratumoral microbiota in immune regulation within pancreatic and other cancers. Nonetheless, the alteration of the tumor immune microenvironment by intratumoral microbiota remains a likely contributing mechanism in pancreatic cancer progression [47].

### 4.4. Anticancer Role of Intratumoral Microbiota

Although intratumoral microbiota can induce tumorigenesis in various ways, in some cases, it can inhibit tumor progression. Indeed, the presence of intratumoral microbiota may have evident local antitumor effects. Different species of gut bacteria have been associated with increased response to immunotherapy. The gut microbiota tends to eliminate cancer cells by assisting the immune system. The intratumoral microbiota can be directly recognized by the host immune system and locally activate the antitumor immunity [44]. Mager LF et al. found that the presence of *Bifidobacterium pseudolongum*, *Lactobacillus johnsonii*, and *Olsenella* significantly improves the effectiveness of immune checkpoint inhibitors in four mouse cancer models. Specifically, *B. pseudolongum* induced a better response to immunotherapy mediated by the increased production of inosine and its translocation through the intestinal barrier, which led to the activation of cytotoxic T cells. This indirect mechanism prompted by both gut and intratumoral microbiota represents a trigger for the stimulation of the A2A adenosine receptor of T cells [114]. Another study performed on patients with metastatic kidney cancer showed that patients presenting a more diversified microbiota and an abundance of *Akkermansia muciniphila* have a better response to immunotherapy [115].

These and other studies demonstrated a well-established modulating effect of gut and intratumoral microbiota on patients’ immune responses. In addition, recent studies have increasingly shown that intratumoral microbiota can also significantly impact the effectiveness of anticancer treatments such as chemotherapy and hormone therapy. In 2023, Wu H and colleagues identified a microbiota signature associated with chemoimmunotherapy response by analyzing the composition of intratumoral microbiota in patients with esophageal cancer. Further investigations on esophageal cancer cell lines and mice models allow the authors to identify intratumoral *Streptococcus* as the main player in the CD8+ T-cell activation and infiltration as well as in the favorable response to chemoimmunotherapy [116]. The improvement of anticancer immune response was also demonstrated by the research group of Singh RP (2023), who demonstrated that the intratumoral composition of OSCC differs depending on the tumor stage with an abundancy of the genera Capnocytophaga, Fusobacterium, and Treponema particularly in the precancer, early cancer, and late cancer stages, respectively. In addition, precancer patients also showed a higher abundance of *Streptococcus* and *Rothia*, thus confirming the results obtained by Wu H. Moreover, they also demonstrated that the local immune response is strictly related to the microbiota composition of the tumor [117].

The anticancer role of intratumoral microbiota was also confirmed in other tumors, including cholangiocarcinoma and liver cancer. Through metagenomics studies on intrahepatic cholangiocarcinoma, Chai and colleagues (2023) identified several abundant bacterial orders in tumors, including Burkholderiales, Pseudomonadales, Xanthomonadales, Bacillales, and Clostridiales. Further studies allowed them to identify *Paraburkholderia fungorum* as a microorganism enriched in paracancerous tissues and negatively correlated with CA19.9 protein levels. In addition, in vitro and animal studies confirmed that *P. fungorum* presents multiple anticancer effects mediating the alteration of amino acid metabolisms and consequently inhibiting the growth of cancer cells [118]. Similarly, microbial investigations performed by NGS techniques on formalin-fixed paraffin-embedded tissue samples of primary liver cancer and matched normal tissue revealed a different tissue-associated microbiota composition in tumor tissues compared to controls. Differences were also observed between tumors with different prognoses, where some bacteria with antitumor effects, including some strains of Pseudomonadaceae, were more abundant in normal tissue or in less aggressive tumors [119].

In a recent case report of a woman with luminal B breast cancer, Vilhais G and colleagues (2024) showed that the efficacy of chemotherapy and endocrine therapies may be influenced by both gut microbiota and intratumoral microbiota, thus influencing the therapeutic outcome of patients. Specifically, the analysis of the patient’s gut microbiota during the treatments evidenced a shift in gut microbial composition from a predominance of Firmicutes to a higher abundance of Bacteroidetes with a consequent loss of microbial diversity. Furthermore, the intratumoral microbiota composition after pharmacological treatments and surgery revealed strong dissimilarity between the microbiota within the residual tumor and the intratumoral microbiota detected in the respective margins, suggesting how both chemotherapy and endocrine therapy affect the composition of intratumoral microbiota [120].

Even though it does not have direct antitumor effects, intratumoral microbiota can be targeted with specific antibiotics for intratumor strains, yielding a dual cytotoxic effect. This approach not only affects the intratumor bacteria associated with neoplastic progression but also impacts the tumor cells [121]. Other approaches are aimed at enriching intratumoral microbiota or at using bacterial metabolites or peptides that showed antitumor effects in vitro; however, more in-depth investigations are needed before considering these supportive treatments effective in cancer patients [122].

### 4.5. Role of Intratumoral Microbiota in Response to Anticancer Drug Treatments

The intratumoral microorganisms play a key role in modulating the effects of anticancer treatments, such as radiotherapy, chemotherapy, and immunotherapy [123,124]. In the context of immunotherapy, an in vivo study performed on 4T1 tumor models demonstrated that the combination of anti-PD-1 immunotherapy treatments and oral Megasphaera sp. XA511 significantly inhibited tumor growth with unknown mechanisms [125]. Similarly, the administration of anti-PD-L1 ICIs and *Bifidobacterium* showed a greater anticancer activity compared to the administration of ICIs alone, suggesting that microorganisms can regulate or enhance the efficacy of immunotherapy [126]. In a study evaluating the anticancer activity of talimogene laherparepvec in melanoma patients, the combination of this drug with pembrolizumab showed a 62% response rate [127]. Therefore, the enrichment of the intratumoral microbiota could favor the activation of the immune system in the TME, sensitizing tumor cells to ICIs. In addition, a promising therapeutic approach for cancer therapy is currently represented by the use of engineered bacteria able to secrete pro-active molecules cooperating with ICIs used in immunotherapy [128]. Regarding the effects of intratumoral microbiota on chemotherapy and radiation therapy, current research is focused mainly on the effects of microorganisms in inducing therapeutic failure or tumor relapse after treatment. Indeed, the TME significantly influences not only the effectiveness of cancer therapies but also plays a central role in driving drug resistance. Combining cytotoxic drugs with strategies to modulate the intratumoral microbiota presents a promising approach to limiting the development of therapy resistance. For example, Gammaproteobacteria and oral pathogens like Aggregatibacter actinomycetemcomitans and P. gingivalis have been shown to produce cytidine deaminase, an enzyme that fully metabolizes gemcitabine, thus contributing to chemoresistance in pancreatic cancer patients [129,130]. In a study using the CT26 mouse model of CRC, intratumoral delivery of E. coli not only altered gemcitabine concentration and efficacy at the tumor site but also impacted the onset of drug resistance [131]. Additionally, in CRC patients, Fusobacterium nucleatum has been associated with oxaliplatin resistance by activating the innate immune response and promoting autophagy [132].

Research on the interaction between intratumoral microbiota and radiotherapy remains limited. However, it has been observed that oral administration of vancomycin-sensitive *Lachnospiraceae* bacteria increases butyric acid levels throughout the body and within tumors, potentially reducing the effectiveness of ionizing radiation [133]. These findings suggest that the presence of *Lachnospiraceae* in the microbiota may impair radiotherapy outcomes.

Furthermore, some studies have investigated the association between tumor-infiltrating bacteria and cancer relapse. For instance, OSCC pathogenesis and progression depend on the modulatory effects played by the oral microbiota. Patients with *F. nucleatum* enrichment showed a lower rate of tumor relapse as well as a reduced metastatic recurrence and a higher progression-free survival and metastasis-free interval [134]. Some studies also evaluate therapeutic interventions based on the administration of bacteria that can infiltrate tumor tissue. In this context, engineered bacteria have been proposed as a strategy for the activation of antitumor immunity through the overexpression of neo-antigens or immune-stimulating factors. In addition, live microorganisms can be used as vectors for tumor vaccination by expressing druggable antigens. [135]. Additionally, the microbiota has the capacity to express immune-dominant T-cell antigens within tumor cells, thereby promoting the presentation of these antigens and initiating memory T-cell responses [128]. Recognition of these tumor-associated antigens activates memory T cells, which then exert cytotoxic effects. Moreover, as tumor cells are destroyed, these antigens can disseminate to neighboring cells, effectively extending antitumor immunity even to areas without direct infection.

### 4.6. Effects of Intratumoral Microbiota in Anticancer Surgery

Surgical resection remains a cornerstone of cancer treatment, particularly for solid tumors. The primary objective of surgery is to remove the tumor mass, which can significantly improve patient prognosis and, in many cases, lead to long-term remission [136]. However, the success of surgery is not solely dependent on the physical removal of the tumor. The biological context, including the presence of intratumoral microbiota, plays a crucial role in influencing surgical outcomes, postoperative recovery, and long-term cancer control.

The presence of pathogenic microorganisms within the tumor can lead to various postoperative complications. Infections at the surgical site are a significant concern, as they can delay wound healing, prolong hospital stays, and increase the risk of morbidity [137]. Bacteria such as *Staphylococcus aureus* and *Pseudomonas aeruginosa* are known to cause postoperative infections, complicating the recovery process [138]. The role of intratumoral microbiota in these infections is increasingly being recognized, as certain bacterial species may colonize the tumor and surrounding tissues, predisposing them to infection following surgical intervention [139].

Additionally, the intratumoral microbiota can impair wound healing by modulating local immune responses and inflammatory pathways [140,141]. For instance, bacteria within the tumor can induce a chronic inflammatory state characterized by the persistent activation of immune cells and the release of inflammatory cytokines. This inflammatory milieu can hinder the normal healing process, leading to delayed closure of surgical wounds and increasing the risk of dehiscence [142]. Understanding the specific microbial populations present within tumors and their contributions to inflammation is crucial for developing strategies to mitigate these postoperative complications.

Beyond immediate postoperative complications, the intratumor microbiota can significantly influence the likelihood of tumor recurrence and metastasis, thus driving the surgical decision-making process [46,143]. Certain microorganisms have been found to create a pro-tumorigenic environment that promotes cancer cell survival and dissemination. *H. pylori*, a bacterium associated with gastric cancer, exemplifies this phenomenon. *H. pylori* infection induces chronic gastritis and a proinflammatory environment, which can lead to genetic mutations and epigenetic changes in gastric epithelial cells, ultimately promoting carcinogenesis and increasing the risk of cancer recurrence after surgical resection [144].

## 5. Diagnostic and Prognostic Role of Intratumoral Microbiota in Cancer

### 5.1. Diagnostic Potential of Intratumoral Microbiota

As described in the previous chapters, specific intratumoral bacteria have been recognized for different tumors. Therefore, researchers worldwide have started to question if the evaluation of intratumoral microbiota can be used for diagnostic purposes [46]. Almost all microbiome-based cancer diagnostics take advantage of sequencing techniques and have been implemented for studies performed on cancers affecting both the respiratory and digestive tracts, including CRC, pancreatic, and lung cancer [63]. However, other tumor types may also be influenced by tumor-specific infiltrating microorganisms that can be used for the detection of specific tumors. Currently, the diagnosis of cancers based on intratumoral microbiota composition is not well validated; however, there are studies aiming to develop a signature of the intratumoral microbiota predictive of the risk of cancer development or to define the prognosis of patients and their response to treatment. The detection of intratumoral bacteria is possible by combining multi-locus sequencing of 16S rRNA amplicon, qPCR, immunohistochemistry, immunofluorescence, culture, and electron microscopy [6]. Histological analyses revealed a different composition of microbes when localized in tumor or immune cells. Through qPCR, the number of bacteria per tissue section was estimated. The diffuse viability of intratumoral bacteria is unclear, especially in tumors with fewer bacteria. However, isolated bacteria were also obtained from breast, lung, prostate, pancreatic, and colon tumors, suggesting diffuse microbial viability [145]. Sepich-Poore and colleagues also analyzed the genomic and transcriptomic data obtained from The Cancer Genome Atlas (TCGA) database to study bacterial, viral, and archaeal nucleic acids in 33 types of cancer.

Given the epidemiological association between colorectal cancer (CRC) and clinical bacteremia, researchers investigated normal samples from the TCGA blood database (n = 1866 samples) to identify cancer-specific microbial DNA. They tested this diagnostic approach by comparing cell-free microbial DNA in plasma from 100 patients with lung, prostate, or melanoma cancers against 69 healthy, HIV-negative individuals. However, limitations exist due to the lack of experimental controls in the TCGA database, dependence on deep sequencing without confirmatory methods, and an unclear understanding of how microbial DNA enters and persists in circulation [145]. A study revealed that in hepatocellular carcinoma (HCC) after surgery, the microbial profiles and community networks between tumors and adjacent normal tissues are markedly different. In particular, it was observed that *Proteobacteria* and *Actinobacteria* were the most abundant phyla in the TME of HCC patients [146]. In head and neck squamous cell carcinoma (HNSC), it was observed that the profiles of tissue-infiltrating microbiota differ between tumor and normal samples. Furthermore, the intratumoral microbiota signature appears to be associated with the clinicopathological features of the tumor (sex, age, tumor stage, and histological grade of the neoplasm), thus serving as an indicator of patients’ prognosis [147].

### 5.2. Prognostic Value of Intratumoral Microbiota

The tumor-infiltrating bacteria may be associated with different survival rates among patients, thus serving as good prognostic indicators. It has been shown that there is a specific microbial signature that could be associated with a poor prognosis in cancer patients. Specifically, the presence of *H. pylori* in gastric carcinoma and CRC contributes to an increased risk of disease and a worse prognosis due to the tumor-promoting effects mediated by the CagA protein [148]. As regards oral cancer or head and neck cancer, the presence of *F. nucleatum* has been associated with EMT, inflammation, and reduced immune response with a consequent worse prognosis [149]. Furthermore, patients with higher levels of *F. nucleatum* had poorer prognosis and a reduced clinical response to anticancer treatments than patients without this bacterium [150]. In contrast, in patients with stage II/III non-MSI-high/non-sigmoid colorectal cancer undergoing oxaliplatin-based adjuvant therapy, the presence of intratumoral *F. nucleatum* has been considered a good independent prognostic factor [151].

In pancreatic cancer, the presence of intratumoral *Proteobacteria* was associated with poor prognosis. *P. gingivalis* was associated with an increased risk of pancreatic cancer. According to Tan Q. et al., *P. gingivalis*-enriched pancreatic cancer showed a faster tumor growth as demonstrated in orthotopic and subcutaneous mouse models of PC. The presence of *P. gingivalis* significantly correlates also with increased inflammation and infiltrating neutrophils. Intratumoral *P. gingivalis* also promotes prostate cancer progression by increasing the secretion of neutrophil chemokines and neutrophil elastase [152]. *Corynebacterium* and *Staphylococcus* spp. could be used as prognostic indicators, as high bacterial load negatively correlates with disease progression and positively with distant metastasis-free survival, overall survival, and T-cell infiltration in nasopharyngeal carcinoma [153]. Pentimalli et al. identified an intratumoral microbiota signature that predicts the prognosis of patients with malignant mesothelioma [154].

Other findings demonstrated differences in the oral microbiota composition observed in patients with oral squamous cell carcinoma (OSCC) compared to normal controls regarding the abundance of the taxa *Firmicutes*, *Actinobacteria*, *Fusobacterium*, *Fusobacteriales*, *Fusobacteriaceae*, and *Fusobacterium*. The presence of all these microorganisms correlated with specific clinical outcomes (survival time and status) and could be proposed for both diagnostic and prognostic purposes [155].

## 6. Conclusions

Emerging lines of evidence show that microorganisms are integral components of the tumor tissue in different cancer types, including CRC, pancreatic, lung, breast, and other cancers. Although cancer tissue was originally thought to be sterile, the discovery of complex microbial signatures within the tumor bulk has led researchers to define and characterize the so-called intratumoral microbiota. Research studies in this context allowed the identification of three main pathways of microbial infiltration in tumor tissues, i.e., the breakdown of mucosal barriers, migration from adjacent normal tissues (NAT), or infiltration through the circulatory system.

The microbes that constitute the intratumoral microbiota have been characterized, and it has been found that they significantly differ depending on the type of tumor; however, the multiple roles that the intratumoral microbiota plays in various cancers have not been fully elucidated yet. Some preliminary findings suggest that within the TME, intratumoral microbiota may play different and contrasting roles in tumor progression. Indeed, the human microbiota is associated with both tumor progression and the formation of metastasis through the production of cytokines, the induction of a proinflammatory response, and the stimulation of the immune response. Some intratumoral bacteria can directly cause DNA damage, enhancing tumor aggressiveness and progression and favoring the metastatization of primary tumors. Such effects are also by the activation of proinflammatory responses and oncogenic pathways or the alteration of the TME. Through all these mechanisms, intratumoral microbiota not only influences tumor growth and metastasis formation but also modulates the efficacy of cancer therapy, potentially inducing anticancer drug resistance.

For all these reasons, the intratumoral microbiota has the potential to be used as both a diagnostic and prognostic tool. Due to the abundance of microorganisms in tumors and the presence of tumor-specific microbial profiles, the evaluation of intratumoral microbiota can be used for diagnostic purposes. Furthermore, several studies have also highlighted a possible prognostic value of the intratumoral microbiota as it correlates with survival rates in different types of cancer.

Finally, besides its diagnostic and prognostic role, intratumoral microbiota and its modulation also have profound effects on patients’ therapeutic response, so much so that they are now considered real modifiers of therapeutic efficacy [156].

In this context, an important aspect to consider is how external microbiomes, such as the gut and oral microbiota, can influence intratumoral microbiota and ultimately impact cancer treatment outcomes. Some studies suggested that microbes from external sources, including the oral cavity and gut, can translocate and become part of the intratumoral microbiota, thus influencing tumor evolution [72]. As widely described in previous chapters, this process occurs through various mechanisms, including bloodstream dissemination, lymphatic migration, or direct translocation from mucosal surfaces.

Maintaining oral hygiene and a balanced intestinal flora are increasingly recognized as critical factors in shaping cancer treatment outcomes. Poor oral hygiene can lead to bacterial infections and systemic inflammation, both of which may hinder the effectiveness of therapies, particularly immunotherapies. For example, oral bacteria like *F. nucleatum*—commonly found in periodontal disease—have been detected in colorectal tumors, potentially migrating from the oral cavity through the bloodstream or gastrointestinal tract [157]. Similarly, gut bacteria, especially under conditions of dysbiosis, may translocate across the intestinal barrier into circulation and eventually colonize tumor tissues.

Once these external bacteria reach the tumor microenvironment, they can alter immune responses, affect the local microbiome, and modulate the efficacy of cancer therapies. The presence of these bacteria in tumors has been associated with altered responses to chemotherapy, immunotherapy, and radiotherapy. Certain bacteria can metabolize chemotherapeutic agents, either reducing their effectiveness or contributing to drug resistance [158]. Among these, *F. nucleatum* has been shown to inhibit apoptosis in cancer cells during chemotherapy, diminishing the treatment’s success. Additionally, the immune-modulating effects of translocated bacteria may influence how well patients respond to immune-based therapies, potentially enhancing or suppressing antitumor immunity [159,160]. Overall, the interplay between external bacteria constituting the human microbiota and the intratumoral microbiota underscores the critical role that maintaining a healthy microbial balance plays in cancer treatment. Efforts to manage oral hygiene and promote gut health, along with strategies like probiotics or fecal microbiota transplants, can help prevent harmful bacterial translocation and support more favorable therapeutic outcomes for cancer patients.

In addition to its diagnostic and prognostic significance, intratumoral microbiota also interacts directly with the human genome, influencing cancer progression and therapeutic responses. More in detail, intratumoral bacteria can modulate gene expression, induce DNA damage, and alter immune responses, ultimately shaping the tumor microenvironment and the effectiveness of treatments [97].

Despite some preliminary findings on this matter, further in vitro and in vivo validation experiments are needed to further understand the interaction between intratumoral microbiota and the human genome. One key mechanism involves the ability of intratumoral bacteria to modulate host gene expression through epigenetic changes, such as DNA methylation and histone modification. These changes can influence genes related to tumor growth, immune response, and treatment sensitivity. For example, *H. pylori*, *Kytococcus sedentarius*, and *Actinomyces oris* induce aberrant DNA methylation, mainly affecting immune genes, thus contributing to the development of stomach adenocarcinoma [161].

Furthermore, some bacteria within tumors can induce genomic instability by generating DNA damage. This occurs either through the production of genotoxins, such as colibactin or through oxidative stress, which generates harmful reactive oxygen species (ROS). These processes can lead to mutations in key genes such as tumor suppressors (including p53), accelerating cancer development and complicating treatment [162]. However, further studies are needed to clearly establish the microorganisms involved in such processes, thus limiting our current understanding of the directly damaging role of intratumoral microbiota.

Finally, intratumoral bacteria may interfere with DNA repair mechanisms, driving a higher mutation rate and resistance to therapies [163], as well as altering the functionality of the immune system by suppressing or enhancing immune activity and the tumor’s ability to evade immune surveillance. For instance, they may upregulate immune checkpoint molecules, dampening the immune response and reducing the effectiveness of immunotherapy [164]. Alternatively, they can stimulate proinflammatory gene expression, improving immune-based treatments by activating antitumor immunity [156].

Despite all these findings, a current limitation of intratumoral microbiota study is related to the fragmented and conflicting data generated on the role of specific bacteria in tumor-promoting and tumor-suppressing processes. In this context, further rigorous in vitro and clinical studies are needed to clarify the role of microbial influences in cancer evolution and treatment.

## Figures and Tables

**Table 1:** Summary of data obtained by Nejman D and colleagues, 2020 [6].

Cancer Type	Bacteria Strains
**Breast cancer**	*Staphylococcus*, *Enterobacteriaceae Comamondaceae*, *Bacteroidetes*, *Proteobacteria*, *Firmicutes*, *Actinobacteria*, *Cyanobacteria*, *Pseudomonas*, *Provetella*, *Propionibacterium*, *Comamonadaceae*, *Gammaproteobacteria*, *Listeria welshimeri*, *Propionibacterium*, and *Brevundimonas*
**Lung cancer**	*Actinobacteria*, *Proteobacteria*, *Firmicutes*, *Corynebacterium*, *Sphingomonas*, *Streptococcus*, *Neisseria*, *Halomonas*, *Kocuria*, *Parvimonas*, *Rothia*, *Veillonella*, *Streptococcus*, and *Bacteroidetes*
**Melanoma**	*Fusobacterium*, *Trueperella*, *Staphylococcus*, *Streptococcus*, *Bacteroides*, *Propionibacterium*, *Corynebacterium*, *Clostridium*, and *Lachnoclostridium*
**Pancreatic cancer**	*Helicobacter*, *Fusobacterium nucleatum*, and *Gammaproteobacteria*
**Ovarian cancer**	*Acinetobacter*, *Sphingomonas*, *Methylobacterium*, *Aquificae*, *Planctomiceti*, *obscuriglobus*, *Halobacteroides halobius*, *Methyloprofundus sedimenti*, *Proteobacteria*, and *Firmicutes*
**Colorectal cancer**	*Morganella morganii*, *Escherichia coli pks+*, *Enterotoxigenic Bacteroides fragilis*, *Fusobacterium nucleatum*, *Enterococcus faecalis*, *Parvimonas micra* and *Peptostreptococcus* spp., *Salmonella* spp., *Clostridium difficile*, *Klebsiella pneumoniae*, *Citrobacter rodentium*, and *Campylobacter jejuni*

## Data Availability

The data reported in this manuscript are available from the corresponding author on request. The original contributions presented in this study are publicly available. These data can be found at the following: https://pubmed.ncbi.nlm.nih.gov/ (accessed on 5 August 2024).
